# Bottom‐up and top‐down effects of tree species diversity on leaf insect herbivory

**DOI:** 10.1002/ece3.2950

**Published:** 2017-04-09

**Authors:** Bastien Castagneyrol, Damien Bonal, Maxime Damien, Hervé Jactel, Céline Meredieu, Evalyne W. Muiruri, Luc Barbaro

**Affiliations:** ^1^ BIOGECOINRAUniv. Bordeaux33610CestasFrance; ^2^ EEFINRAUniversité de Lorraine54280ChampenouxFrance; ^3^ ECOBIOUMR CNRS 6553Université de Rennes35042RennesFrance; ^4^ School of Biological SciencesRoyal Holloway University of LondonEghamSurrey TW20 0EXUK; ^5^ Department of BiosciencesDurham UniversityStockton Road, DurhamDH1 3LEUK; ^6^ DynaforINPTEI PurpanINRAUniversité de Toulouse31320AuzevilleFrance

**Keywords:** associational effects, biodiversity, drought, dummy caterpillars, leaf traits, predation, *Quercus robur*

## Abstract

The diversity of plant neighbors commonly results in direct, bottom‐up effects on herbivore ability to locate their host, and in indirect effects on herbivores involving changes in plant traits and a top‐down control by their enemies. Yet, the relative contribution of bottom‐up and top‐down forces remains poorly understood. We also lack knowledge on the effect of abiotic constraints such as summer drought on the strength and direction of these effects. We measured leaf damage on pedunculate oak (*Quercus robur*), alone or associated with birch, pine or both in a long‐term tree diversity experiment (ORPHEE), where half of the plots were irrigated while the other half remained without irrigation and received only rainfall. We tested three mechanisms likely to explain the effects of oak neighbors on herbivory: (1) Direct bottom‐up effects of heterospecific neighbors on oak accessibility to herbivores, (2) indirect bottom‐up effects of neighbors on the expression of leaf traits, and (3) top‐down control of herbivores by predators. Insect herbivory increased during the growth season but was independent of neighbor identity and irrigation. Specific leaf area, leaf toughness, and thickness varied with neighbor identity while leaf dry matter content or C:N ratio did not. When summarized in a principal component analysis (PCA), neighbor identity explained 87% of variability in leaf traits. PCA axes partially predicted herbivory. Despite greater rates of attack on dummy caterpillars in irrigated plots, avian predation, and insect herbivory remained unrelated. Our study suggests that neighbor identity can indirectly influence insect herbivory in mixed forests by modifying leaf traits. However, we found only partial evidence for these trait‐mediated effects and suggest that more attention should be paid to some unmeasured plant traits such as secondary metabolites, including volatile organic compounds, to better anticipate the effects of climate change on plant‐insect interactions in the future.

## 1 Introduction

Plant diversity is a key driver of insect herbivory in grassland, agricultural, and forest ecosystems (Allan et al., 2013; Broad, Schellhorn, Lisson, & Mendham, 2008; Castagneyrol et al., 2014), because heterospecific neighbors may either decrease (associational resistance) or increase (associational susceptibility) the likelihood of focal plants being attacked by insect herbivores (Barbosa et al., 2009; Root, 1973; Tahvanainen & Root, 1972; White & Whitham, 2000). Several reviews and meta‐analyses suggest that associational resistance is the most common pattern (Andow, 1991; Barbosa et al., 2009), particularly in forest ecosystems (Castagneyrol et al., 2014; Jactel & Brockerhoff, 2007). However, despite the large body of evidence on associational effects (Moreira et al., 2016; Underwood, Inouye, & Hambäck, 2014), gaps in our understanding of underlying mechanisms hinder the development of a predictive framework of herbivore responses to tree diversity. In addition, due to the rising threats to forests that result from climate change, like more severe or frequent outbreaks of pest insects (Logan, Régnière, & Powell, 2003), it is critical to better evaluate the relative contribution of factors responsible for associational resistance or susceptibility under contrasting abiotic constraints.

Associational effects depend on the identity of heterospecific neighbors, both directly through bottom‐up effects on focal host accessibility (e.g., Castagneyrol, Giffard, Péré, & Jactel, 2013) and indirectly through changes in focal host quality (e.g., Kos, Bukovinszky, Mulder, & Bezemer, 2015; Kostenko, Mulder, Courbois, & Bezemer, 2016; Mraja, Unsicker, Reichelt, Gershenzon, & Roscher, 2011) and top‐down control of herbivore populations by predators (e.g., Muiruri et al. 2015). However, the relative importance of top‐down and bottom‐up effects remains unclear, in particular because few studies to date addressed them simultaneously (Abdala‐Roberts et al., 2016; Moreira, Mooney, Zas, & Sampedro, 2012).

Three alternative mechanisms have been proposed to explain associational resistance. First, associational resistance has been proposed to result primarily from a reduced ability of herbivores to locate and reach their host plants among heterospecific neighbors. This may be due to patches with greater plant diversity being less attractive than monospecific patches (i.e., the resource concentration hypothesis, Root, 1973; Andersson, Löfstedt, & Hambäck, 2013) or from heterospecific neighbors reducing the physical (Castagneyrol et al., 2013; Damien et al., 2016; Floater & Zalucki, 2000) or chemical (Jactel, Birgersson, Andersson, & Schlyter, 2011; Zhang & Schlyter, 2004) apparency of host plants. Alternatively, the attractant‐decoy hypothesis predicts that herbivores can be diverted from a given plant and aggregate on more apparent, more attractive, or more palatable neighbors (Atsatt & O'Dowd, 1976; Hahn & Orrock, 2016).

Second, heterospecific neighbors may affect insect herbivory on a focal plant through changes in host food quality, although this is less well documented (but see, e.g., Walter et al., 2011; Kos et al., 2015). Because the amount of insect herbivory depends on plant traits such as leaf toughness, water content, C:N ratio, and secondary metabolites (Loranger et al., 2013; Moreira, Abdala‐Roberts, Parra‐Tabla, & Mooney, 2014; Pearse, 2011), any biotic or abiotic factor affecting the expression of these plant traits may also affect herbivores. For instance, competition or facilitation among the focal plant and its neighbors may change the nutritional value of the host plant tissues (Kos et al., 2015; Schädler, Brandl, & Haase, 2007; Walter et al., 2011) or its production of secondary metabolites to defend against herbivores (Moreira et al., 2014).

Third, the enemies’ hypothesis posits that predators and parasitoids of herbivores are more abundant and diverse in species‐rich plant communities (Elton, 1958; Root, 1973; Schuldt et al., 2011; Straub et al., 2014). Associational resistance may therefore also result from an enhanced top‐down control of herbivores by predation or parasitism in species‐rich plant communities (Riihimäki, Kaitaniemi, Koricheva, & Vehviläinen, 2004). Besides its effect on abundance and richness of enemies, plant diversity may indirectly influence the strength of herbivory suppression by predators. For instance, greater predation is expected when herbivores are more exposed to their enemies as they spend more time foraging for less accessible resource (Straub et al., 2014). Plant diversity may also modify the magnitude of top‐down effects by altering the proportion of generalist versus specialist herbivores because herbivore communities dominated by generalist herbivores are more sensitive to predation (Singer et al., 2014).

The strength and direction of plant‐herbivores‐enemies (predators or parasitoids) interactions is expected to change along environmental gradients (Bauerfeind & Fischer, 2013; Péré, Jactel, & Kenis, 2013; Rodríguez‐Castañeda, 2013; Walter et al., 2011). Rooted in the plant stress hypothesis (White, 1974) that predicts increasing plant susceptibility to herbivores with higher water stress, several studies suggest that drought favors leaf‐feeding herbivores and reduces damage caused by sap‐feeders (Huberty & Denno, 2004; Jactel et al., 2012). In particular, drought might affect the palatability of plant tissues, notably through change in carbohydrate content and C:N ratio (Jactel et al., 2012; Walter et al., 2011). In addition to changes in leaf traits interfering with plant quality, drought may also indirectly affect herbivory by modifying predator attraction (Aslam, Johnson, & Karley, 2013; Staley et al., 2006; Weldegergis, Zhu, Poelman, & Dicke, 2015). Yet, although underlying mechanisms remain unclear, it is increasingly acknowledged that neighbors can also modify plant's response to drought (Forrester, Theiveyanathan, Collopy, & Marcar, 2010; Grossiord et al., 2014). Insect herbivory may therefore be affected by both plant diversity and water stress, as well as their potential interacting effects (Walter et al., 2011).

Experimental evidence that plant neighborhood mediates the effect of water stress on herbivores and their enemies—or the opposite—is still lacking given the difficulties to control for both the composition of tree neighborhood and climatic variables. In this study, we searched for mechanisms responsible for associational effects under contrasting abiotic conditions. We measured insect herbivory as the percentage of defoliation by chewing herbivores on pedunculate oak (*Quercus robur*) in a tree diversity experiment with a factorial design crossing the identity of their neighbors (homospecific vs. heterospecific) with an irrigation treatment (rainfall only vs. rainfall *plus* irrigation). We specifically addressed mechanisms underlying neighbors and drought effects on insect herbivory. We predicted that: (1) herbivory is greater in tree monocultures where individual oaks are more apparent than in tree mixtures where they are protected by taller nonoak neighbors, (2) tree neighbors indirectly mediate the expression of traits involved in herbivore‐oak interaction; (3) heterospecific neighbors increase top‐down predation by natural enemies, and (4) water stress changes the strength of top‐down and bottom‐up processes. Overall, this study addresses how drought indirectly mediates tree neighbor effects on insect herbivory via changes in leaf traits and top‐down control by herbivores’ enemies. We provide partial support to the hypothesis of indirect, bottom‐up, effect of tree species diversity on herbivory through changes in leaf traits, regardless of drought.

## 2 Materials and Methods

### 2.1 Ethics statement

This study did not involve manipulations of humans or animals. No specific permissions were required for our field work. The study did not involve endangered or protected species.

### 2.2 The ORPHEE experiment

This study was carried out in the ORPHEE experiment in SW France (44°440 N, 00°460 W). The design has been fully described in Castagneyrol et al. (2013), and we will thus only provide here a brief overview. In 2008, eight blocks were established covering 12 ha, with 32 plots in every block corresponding to the 31 possible combinations of one to five tree species (*Betula pendula*,* Quercus robur*,* Q. pyrenaica*,* Q. ilex,* and *Pinus pinaster*) with an additional replicate of the five‐species plot. Each plot contained 10 rows of 10 trees planted 2 m apart (100 trees). Tree species mixtures were established according to a substitutive design, keeping tree density and the identity of tree neighbors equal across plots. Within plots, individual trees from different species were planted in a regular alternate pattern, such that a tree from a given species had at least one neighbor from each of the other species within a 2‐m radius (Fig. S1 and www.facebook.com/orpheeexperiment).

In this study, we focused on a subsample of plots to reconstruct a tree diversity gradient spanning from the monoculture of pedunculate oak, the two‐two‐species mixtures associating oak with pine or birch, and the three‐species mixtures of oak, birch, and pine, for a total of four compositions. These plots were chosen in order (1) to span a gradient of oak apparency by increasing the number of neighbors taller than oaks (i.e., high apparency in monocultures, intermediate apparency in oak‐birch and oak‐pine mixtures and low apparency in oak‐birch‐pine mixtures, Castagneyrol et al., 2013) and (2) to contrast functional diversity by associating oak with a broadleaved or a conifer species. Within the selected 32 plots (4 plots × 8 blocks), we sampled at random six individual trees of the 36 innermost trees of each plot, for a total of 192 sampled oaks.

In 2015, half of the blocks were irrigated (Fig. S2). Irrigation consisted in sprinkling *ca* 42 m³ per night and per block from early May to late September, corresponding to *ca* 3 mm/day per plot. This volume was calculated based on regional climatic data (evapotranspiration) and was assumed to avoid any soil water deficit in the irrigated blocks during the entire growing season.

### 2.3 Insect herbivory

Insect herbivory was assessed twice to test for a season effect, in early June and early August 2015 by visual inspection of 30 leaves per sampled oak (Johnson, Bertrand, & Turcotte, 2016). Three branches were selected at the top, middle, and bottom of each tree. Five leaves were then randomly chosen at the tip, and five at the basis of each branch. The percentage of leaf area removed (LAR) by insect defoliators (chewers and skeletonizers) was estimated on each sampled leaf by a unique observer all along the experiment (BC) using seven classes (0%, 1%–5%, 6%–15%, 16%–25%, 26%–50%, 51%–75% and >75% LAR) and then averaged per sampled tree using the midpoint of each damage class.

### 2.4 Leaf traits

We measured five leaf traits known to significantly influence insect herbivory: specific leaf area (SLA), leaf dry matter content (LDMC), leaf toughness and thickness, and leaf C:N ratio (e.g., Loranger et al., 2013; Pearse, 2011).

Leaf traits may vary in response to the identity of oak neighbors, herbivory, or both. To avoid confounding the effect of tree species neighbors and insect herbivory on leaf traits, half of the sampled trees were sprayed every two weeks with a broad action spectrum insecticide (5% λ‐cyhalothrin, KARATE^®^, Syngenta, diluted at 15 g/hl). Sprayed and unsprayed oaks were at least 4 m apart to reduce accidental drift of insecticide onto control oaks.

In June 2015, we randomly collected six sunlit, mature, and fully expanded leaves from the top part of the canopy on each target oak (*n* = 6 per plot, for a total of 192 trees), stored them in zipped plastic bags and immediately put them into a cool box. In order to standardize trait measurements, leaves were rehydrated for 48 hr (Pérez‐Harguindeguy et al., 2013) and then scanned and weighted individually. Leaf toughness was assessed as the resistance to piercing, with six measurements per leaf. Measurements were conducted with a dial tension gauge model with peak hold (Mitutoyo Messgeräte Leonberg GmbH, Leonberg, Germany). Leaves were then dried until constant weight (at least 48 hr at 60°C) and weighted again to obtain LDMC (mg/g) and SLA (cm^2^/g). Leaf thickness was derived from SLA and LDMC (Pérez‐Harguindeguy et al., 2013). In early August 2015, we again collected six leaves per tree as explained above. Leaves were dried and then bulked for each tree and finely grinded. Leaf carbon isotope composition (δ^13^C, per mil), foliar carbon (C%, %), and nitrogen (N%, %) contents were measured on these bulked samples. Isotope and elementary analyses were performed at the INRA Nancy Technical Facility of Functional Ecology (OC 081) with an EA/GA‐IRMS (Carlo Erba, Elementar, Finnigan, Isoprime, Bremen, Germany). C% and N% were used to calculate C:N ratios. Together with the punctual measurement of leaf water potential (see below), δ^13^C was used to confirm that irrigation actually alleviated water stress.

### 2.5 Tree water status

To assess the effectiveness of irrigation at alleviating water stress, we compared tree water status of oak trees by measuring predawn leaf water potential (ψ_w_) in early August 2015 on 1–3 oaks per plot. ψ_w_ is used as a proxy of the spatially integrated water potential of the soil explored by roots. We used a Scholander‐type pressure chamber (model 1000; PMS Instruments, Corvalis, OR) to measure Ψ_w_ on one leaf per oak tree. Leaves were sampled between 4:30 and 6.30 a.m. local time (before sunrise) with clippers.

### 2.6 Insectivory

Total and avian insect predation rates were assessed using a standardized method (e.g., Barbaro et al., 2014; Mäntylä et al., 2008; Muiruri, Rainio, & Koricheva, 2016). We modeled 2‐cm‐long dummy caterpillars made with green modeling clay to mimic larvae of locally abundant moth species, notably Geometridae and Noctuidae. Dummy caterpillars were secured on oak branches with a thin metallic wire. Three to six oaks were randomly selected at the center of each sampled plot. We installed three caterpillars per oak, at the tip of three different branches at *ca* 1.5 m from the ground. Insectivory was assessed twice, after 3 and 6 weeks of exposure (June 9–10 and July 2) by counting the number of lures showing obvious beak (birds), teeth (micro‐mammals), and mandibles (arthropods) marks per individual tree.

### 2.7 Analyses

Irrigation being applied at the block level (i.e., *whole plot*), the ORPHEE experiment is a split‐plot experiment which requires adapting the calculation of degrees of freedom and mean sum of squares of residuals. This was achieved using linear mixed effect models (LMM), with *Block* and *Block* × *Irrigation* as random factors (1|*Block*:*Irrigation* in *R* syntax). Herbivory and leaf trait data were analyzed at the tree level. Nonindependence of individual oaks within plots was accounted for by defining plot as a random factor, nested within blocks (Schielzeth & Nakagawa, 2013). For each test, we first built a full model including all fixed effects and their interactions (see details for each response variable below). The full model was then simplified by sequentially dropping nonsignificant terms, starting with highest‐order interactions. Model parameters were finally estimated on the most parsimonious model obtained using restricted maximum likelihood (REML).

### 2.8 Leaf traits

Leaf traits were measured on all trees, insect damage might trigger induced defenses modifying leaf traits. To test the direct, independent effect of oak neighbor identity (here after “‘*Neighbors”*) on leaf traits, we limited the analysis of tree diversity on leaf traits to insecticide sprayed trees. The insecticide treatment reduced herbivory by *ca* 33% in spring (Kruskal‐Wallis test: χ² = 40, *p* < .0001) and 45% in summer (Kruskal‐Wallis test: χ² = 50, *p* < .0001). Traits were analyzed individually, using the same LMM modeling approach, with *Irrigation*,* Neighbors,* and their interaction as fixed effects.

Then, we tested whether oak leaf traits could account for variability in herbivory. We conducted a principal component analysis (PCA) on traits measured on unsprayed trees and extracted coordinates on the two‐first PCA axes (PC1 and PC2, respectively) that together explained 88% of variability in leaf traits. We used LMM (see above) to test whether PC1 and PC2 were explained by *Irrigation*,* Neighbors,* or their interaction.

### 2.9 Insect herbivory

Only unsprayed trees were included in this set of analyses. We first tested the effect of oak neighbors and irrigation on insect herbivory using the same approach as for traits. Fixed effects were *Season*,* Neighbors,* and *Irrigation*, and all interactions. Because herbivory was assessed twice on the same individual oaks, we added tree identity as a random factor, nested within *Block* and *Plot* factors, to account for repeated measurements.

We then tested whether the effect of neighbors could be accounted for through changes in leaf traits measured on the same trees or predation pressure at the plot scale (i.e., proportion of attacked caterpillars pooled across the two surveys). *Traits* and *Predation* were included into the model as fixed effects, together with *Irrigation* and *Neighbor* factors and *Neighbors *× *Irrigation* interactions. Because trait values may have changed during the course of the season, and because predation was only assessed in early summer, we tested the effects of SLA, LDMC, leaf toughness, thickness, and predation on early season herbivory, and the effect of C:N on late season herbivory, separately. Given that both leaf traits and predation rates might have been influenced by irrigation and neighbor identity, this approach allows testing whether some residual variance can still be explained by plot‐level factors (i.e., *Irrigation* and *Neighbors*) when accounting for traits and predation, and conversely. We expected that, should herbivory be better explained by leaf traits or predation than by irrigation or neighbor identity, significant effects of any of these factors (or their interaction) would become nonsignificant once traits and predation are accounted for. Conversely, should irrigation or neighbor identity effects remain significant after including leaf traits and predation, this would suggest that irrigation and neighbor identity influence herbivory through other unmeasured plant traits or unknown natural enemies (e.g., parasitoids).

Finally, we summarized the univariate effects of all individual traits using coordinates on the two PCA axes and followed the same approach to model insect herbivory as a function of *PC1*,* PC2*,* Irrigation*,* Neighbors*,* PC1 *×* PC2,* and *Irrigation* × *Neighbors* interactions. We then applied the same simplification procedure as explained above.

### 2.10 Insectivory

We analyzed predation rates using generalized LMM (GLMM), with binomial error and logit link. The response variable was the number of attacked versus nonattacked dummy caterpillars per tree. We modeled separately the response of total predation (i.e., all predators combined, including birds, small rodents, and arthropods) and avian predation only to neighbor identity and irrigation.

For every model, conditions of application were visually checked and the response variable was log‐transformed whenever necessary. All analyses were conducted in R (R Core Team, 2016) using the *lmerTest* and *ade4* libraries.

## 3 Results

### 3.1 Effects of irrigation and neighbors on water stress in oaks

Predawn leaf water potential of oaks (ψ_w_) was significantly lower (*F* = 35.13, *p* < .0001) in blocks receiving only rainfall (−0.37 MPa) than in irrigated blocks (−0.19 MPa), confirming the effectiveness of the irrigation treatment (Fig. S1). There was a significant effect of plot composition on ψ_w_ in oaks (*F* = 4.14, *p* = .017), with lower ψ_w_ in nonirrigated plots containing pines (Fig. S1). Consistently, δ^13^C was greater in the mixture with pine as compared to the two other mixtures and monocultures, thus confirming the greater water stress of oaks in two‐ and three‐species mixtures including pine (Figure 2).

### 3.2 Effects of irrigation and oak neighbor identity on insect herbivory

**Figure 1:**
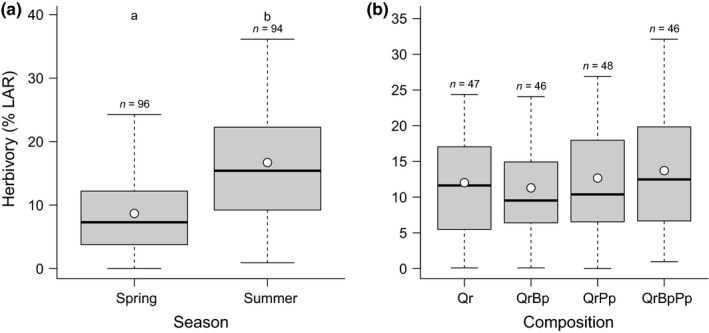
Effects of season (a) and tree species composition (b) on herbivory (% Leaf Area Removed—%LAR). Rainfall only and irrigated plots are combined. Boxes indicate the lower and upper quartiles. Thick horizontal lines and dots represent the median and the mean, respectively. Different letters above boxes indicate significant differences. *n* indicates sample size. Qr, *Quercus robur*; QrBp, *Q. robur *+ *Betula pendula*; QrPp, *Q. robur *+ *Pinus pinaster*; QrBpPp, *Q. robur *+ *B. pendula *+ *P. pinaster*

On average (±*SE*), insect herbivory on unsprayed trees was twice as high in summer compared to spring (16.7 ± 1.0 in summer and 8.7 ± 0.6% leaf area damaged in spring, respectively, *F*_(1,92.4)_ = 78.72, *p* < .001, Figure 1a).

Insect herbivory did not vary with the identity of oak neighbors (*F*_(3, 85.1)_ = 0.60, *p* = .617, Figure 1b), regardless of the season (*Neighbors* × *Season*:* F*_(3,89.8)_ = 1.58, *p* = .201).

The effect of irrigation was not significant (*F*_(1,6.0)_ = 0.01, *p* = .913), neither separately nor in interaction with the season (*F*_(1,88.8)_ = 0.50, *p* = .479) nor with oak neighbor identity (*F*_(3,81.9)_ = 0.72, *p* = .543). The simplified model only retained the season as predictor and explained 21% of variability in herbivory (*R* ^2^_m_ = 0.21, *R* ^2^_c_ = 0.49).

### 3.3 Effects of irrigation and neighbor identity on oak leaf traits

**Figure 2:**
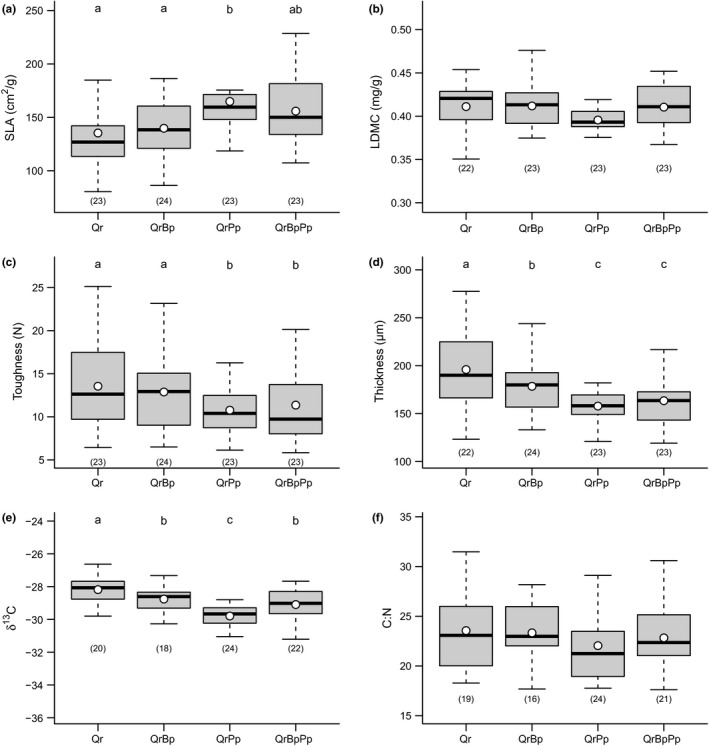
Effects of tree species composition on oak leaf traits. (a) Specific leaf area, (b) leaf dry matter content, (c) leaf toughness, (d) leaf thickness, (e) leaf δ^13^C (×−1), (f) Leaf C:N. Data were summarized across all blocks, regardless of irrigation. Traits were compared in trees sprayed with insecticide such that differences in leaf traits are independent of any herbivory effect. Boxes indicate the lower and upper quartiles. Thick horizontal lines and dots represent the median and the mean, respectively. Same letters above bars indicate nonsignificant differences. Number within brackets below boxes indicates sample size. Qr, *Quercus robur*; QrBp, *Q. robur *+ *Betula pendula*; QrPp, *Q. robur *+ *Pinus pinaster;* QrBpPp, *Q. robur *+ *B. pendula *+ *P. pinaster*

In sprayed oak trees, SLA, leaf toughness, and leaf thickness significantly varied with oak neighbor identity (Figure 2), but none was influenced by irrigation or *Irrigation *× *Neighbor* interaction (Table 1). SLA was higher in plots where oak was associated with pine (two‐ and three‐species mixtures). Toughness was lower in plots where pine was present (two‐ and three‐species mixtures). Thickness was higher in monocultures than in mixed plots. There was no significant effect of oak neighbor identity on LDMC or C:N.

**Table 1:** Summary of LMM testing the effects of oak neighbor identity and block irrigation on oak leaf traits

Trait	Predictors	*F* value (*df*)	*p* value	*R* ^2^m (*R* ^2^c)
SLA	Neighbors	4.20 (3, 20.73)	**.018**	0.12 (0.29)
	Irrigation	0.01 (1, 7.36)	.91	
	Neighbors × irrigation	1.37 (3, 18.04)	.283	
LDMC	Neighbors	1.77 (3, 20.26)	.185	– (0.30)
	Irrigation	0.01 (1, 6.49)	.916	
	Neighbors × irrigation	2.81(3, 17.21)	.07	
Toughness	Neighbors	5.08 (3, 81.66)	**.003**	0.05 (0.70)
	Irrigation	0.19 (1, 8.00)	.672	
	Neighbors × irrigation	0.45 (3, 78.50)	.715	
Thickness	Neighbors	13.21 (3, 81.91)	**<.0001**	0.22 (0.56)
	Irrigation	0.02 (1, 7.44)	.901	
	Neighbors × irrigation	0.46 (3, 78.63)	.711	
δ13C	Neighbors	14.97 (3, 18.71)	**<.0001**	0.35 (0.62)
	Irrigation	4.65 (1, 5.93)	.075	
	Neighbors × Irrigation	0.64 (3, 15.46)	.6	
C:N	Neighbors	0.49 (3, 19.16)	.695	−(0.34)
	Irrigation	0.02 (1, 6.18)	.884	
	Neighbors × irrigation	0.65 (3, 17.04)	.591	
PC1	Neighbors	13.17 (3, 19.32)	**.0001**	0.34 (0.65)
	Irrigation	0.11 (1, 5.86)	.748	
	Neighbors × irrigation	1.49 (3,15.30)	.256	
PC2	Neighbors	0.90 (3, 57.80)	.0447	−(0.38)
	Irrigation	0.07 (1, 6.00)	.794	
	Neighbors × irrigation	0.07 (3, 54.70)	.978	

Marginal (*R* ^2^_m_) and conditional (*R* ^2^_c_) *R* ^2^ were calculated for the simplified model. They correspond to the variance explained by fixed and fixed *plus* random factors, respectively.

Bold values indicate significant effects (*P* < 0.05).

**Figure 3:**
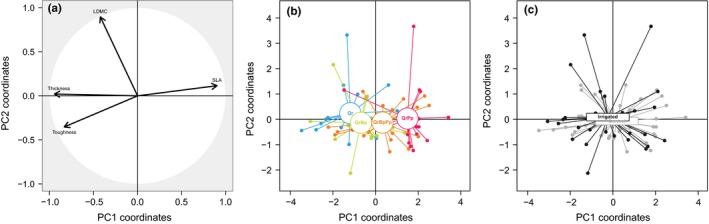
Principal Components Analysis of leaf traits. (a) Correlation circle showing correlations among leaf traits (SLA: specific leaf area; LDMC: leaf dry matter content) and between leaf traits and PCA axes. (b‐c) Projections of individual trees on PCA axes according to (b) tree species composition and (c) irrigation treatment. Qr, *Quercus robur*; QrBp, *Q. robur *+ *Betula pendula*; QrPp, *Q. robur *+ *Pinus pinaster*; QrBpPp, *Q. robur *+ *B. pendula *+ *P. pinaster*

The first and second axes of the principal component analysis explained 64.3% (PC1) and 23.5% (PC2) of variability in leaf traits, respectively. PC1 was driven by SLA, thickness, and toughness, positive values being associated with large, thin, and soft leaves (Figure 3a). PC2 was driven by water content, positive values being associated with high LDMC, that is, low water content (Figure 3a). There was a significant effect of oak neighbors on PC1, which was independent of irrigation (Table 1). Oak leaf traits were not significantly different between monocultures and oak‐birch mixtures, but with two‐ and three‐species mixtures containing pine (Figure 3b). There was no effect of neighbors or irrigation on PC2.

### 3.4 Effects of irrigation and oak neighbors on predation

**Figure 4:**
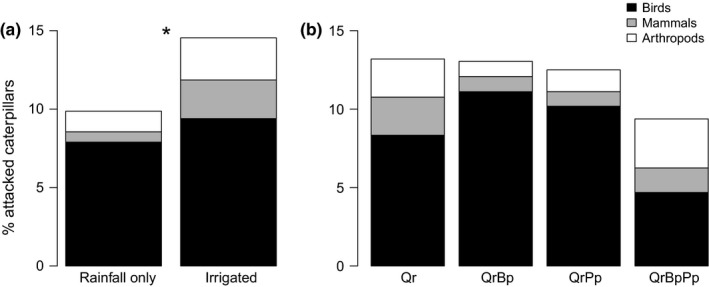
Effects of block irrigation (a) and tree species composition (b) on predation (i.e., attacks of dummy caterpillars). ‘*’ indicates significant difference between treatments. Qr, *Quercus robur*; QrBp, *Q. robur *+ *Betula pendula;* QrPp, *Q. robur *+ *Pinus pinaster*; QrBpPp, *Q. robur *+ *B. pendula *+ *P. pinaster*

Overall, the predation rate of dummy caterpillars averaged across predator identity, plots, and dates was 12.7 ± 1.7%. Among the 110 lures displaying obvious predator marks (of 1,359 observations), 70.9% were attributed to birds (Figure 4). Attacks by small rodents (16.4%) and arthropods (12.7%) were too scarce and unevenly distributed to be analyzed separately. Insectivory was thus analyzed first for all predators and then for birds only. Overall, total insectivory was higher in irrigated than in rainfall only plots (χ² = 4.62, *p *= .032, Figure 4). Differences in predation rates among plots with different tree species composition were not significant (χ² = 1.89, *p *=* *.596, Figure 4). There was no difference between the two surveys (χ² = 0.12, *p* = .728), and results were identical when only bird insectivory was considered. There was no correlation between the proportion of predated dummy caterpillars and the percentage of leaf area removed by herbivores, neither at the tree (Pearson's *r *=* *−0.04, *p *=* *.814) nor at the plot scale (Pearson's *r *=* *0.13, *p *=* *.100).

### 3.5 Effects of oak leaf traits on insect herbivory

**Figure 5:**
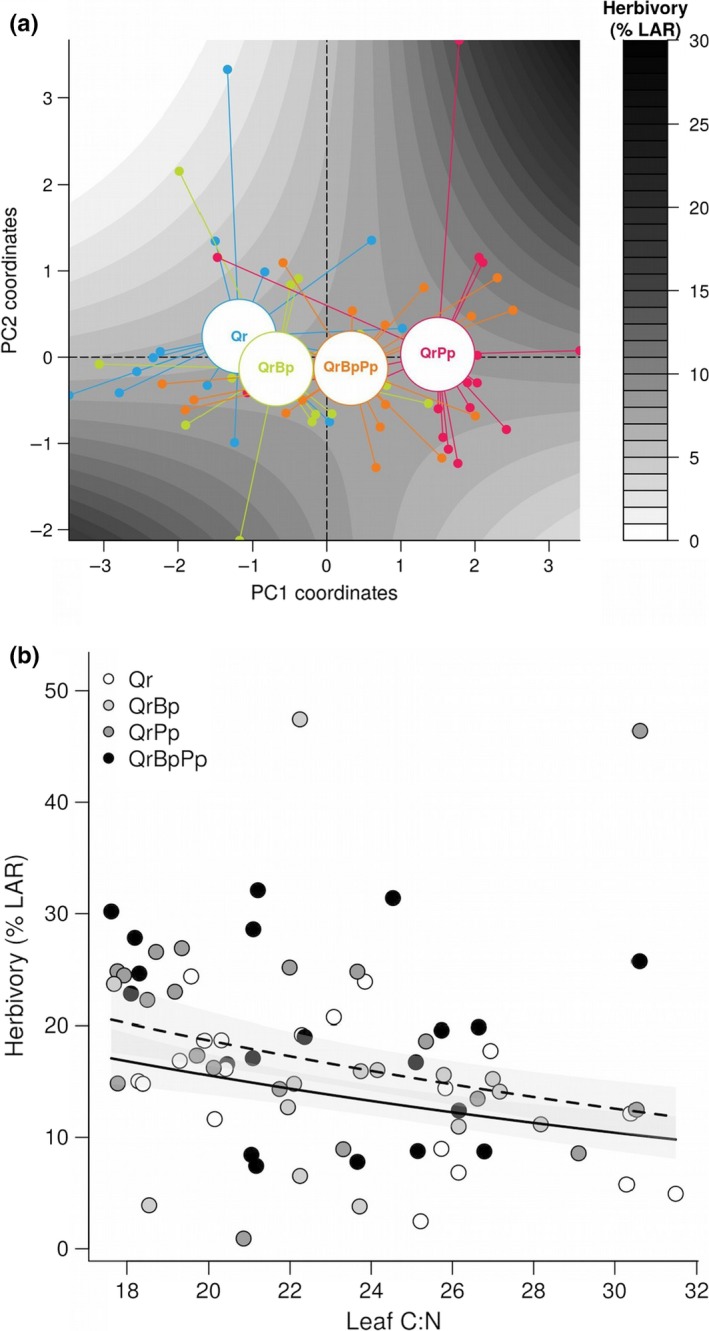
Effect of leaf traits on herbivory. (a) Interactive effects of PC1 and PC2 on herbivory. Gray scale colors represent herbivory (% leaf area removed—%LAR) predicted by GLMM retaining only PC1, PC2, and their interaction as predictors. Individual trees are plotted as in Figure 3b. Herbivory increased strongly with PC1 for high values of PC2, but decreased slightly with PC1 for low values on PC2. Herbivory increased along PC2 axis when PC1 values were high, while it decreased slightly for low values of PC1. (b) Effects of C:N ratio on late season herbivory. Dashed and solid regression lines are for irrigated and rainfall only blocks, respectively. Shaded area represent model *SE*. Qr, *Quercus robur*; QrBp, *Q. robur *+ *Betula pendula*; QrPp, *Q. robur *+ *Pinus pinaster*; QrBpPp, *Q. robur *+ *B. pendula *+ *P. pinaster*

When considered individually, none of the leaf traits measured in early season (i.e., SLA, thickness, toughness, LDMC) on unsprayed oak trees had a significant effect on insect herbivory (Table S1). Insectivory had no effect on herbivory neither (Table S1). While individually, the measured traits failed to explain variability in insect herbivory, there was a significant effect of *PC1 *×* PC2* interaction on early season herbivory (*F*_(1,57.6)_ = 5.30, *p* = .025). Although main effects of PC1 and PC2 were not significant, model parameter estimates were positive for PC1 (0.08 ± 0.09) and negative for PC2 (−0.11 ± .16). Furthermore, the significant interaction between PC1 and PC2 had a positive parameter estimate (model parameter estimate ± *SE*: 0.20 ± 0.09) which indicates that herbivory tended to increase more along PC1 axis (i.e., with larger, thinner, and smoother leaves) when PC2 values were high (i.e., leaves with a low water content) and conversely (Figure 5a). These results indicate that altogether, leaf trait variation can significantly contribute to explain insect herbivory, although the final model explained only a small fraction (8%) of variability in early season herbivory (*R* ^2^_m_ = 0.08, *R* ^2^_c_ = 0.25).

In late season, insect herbivory significantly decreased with the C:N ratio of oak leaves (*F*_(1,66.5)_ **= 4.53, *p* = .037, Figure 5b), irrespective of irrigation (*Irrigation*:* F*_(1,29.4)_ = 1.38, *p* = .249; *Irrigation* × C:N: *F*_(1,62.6)_ = 0.70, *p* = .405). C:N ratio, however, only explained 6% of variability in late season herbivory (*R* ^2^_m_ = 0.06, *R* ^2^_c_ = 0.19).

## 4 Discussion

Contrary to our expectations (i.e., associational effects in presence of heterospecific neighbors), insect herbivory on oak was not influenced by oak neighbor identity. Insect herbivory was, however, partially influenced by leaf traits, which responded to the identity of oak neighbors. Despite higher predation in irrigated plots, water stress had no direct or indirect effect on insect herbivory.

### 4.1 Oak neighbor identity had weak indirect trait‐mediated effects on insect herbivory

Contrary to previous studies reporting an effect of tree composition or diversity on insect herbivores associated with oaks (Alalouni, Brandl, Auge, & Schädler, 2014; Castagneyrol et al., 2013; Setiawan et al., 2014), we observed no difference in insect herbivory among different neighbor identities. Two main mechanisms have been proposed to explain why herbivory should vary with the composition of forests: (1) The probability of a tree being colonized by herbivores (i.e., its apparency, frequency, and concentration) may depend on the diversity and identity of its neighbors (Barbosa et al., 2009; Castagneyrol et al., 2013; Hambäck, Inouye, Andersson, & Underwood, 2014; Setiawan et al., 2016) and (2) the abundance and activity of herbivore enemies may change with forest composition and structure (Muiruri et al., 2016; Riihimäki et al., 2004; Schuldt et al., 2011). Our results support neither of these. Oak apparency, frequency, and concentration (i.e., oak accessibility) were consistently low in all mixtures as compared to monocultures, and yet, they did not receive lower herbivory in the presence of taller birches or pines (Castagneyrol et al., 2013). It is unlikely that the effects of greater apparency on herbivore recruitment were compensated by higher predation rates in oak monocultures, as predation remained unaffected by oak neighbor identity.

Here, we additionally investigate a third, complementary hypothesis, where (3) oak neighbor identity could modify leaf traits involved in tree‐herbivore interactions (Halpern, Bednar, Chisholm, & Underwood, 2014; Kos et al., 2015). Our results partially support this hypothesis.

#### 4.1.1 Oak leaf traits varied with oak neighbor identity

The main differences in oak leaf traits were detected between oak monocultures and mixtures (Figure 2), in particular between monocultures and oak‐pine mixtures. In the ORPHEE experiment, in 2015, oaks (mean height ± *SE*: 110.0 ± 0.8 cm) were much smaller than birches (510.0 ± 2.0 cm) and pines (563.0 ± 1.5 cm) (Damien et al., 2016). The total amount of light received by oaks was thus likely reduced in mixed plots where oaks were dominated by pines and birches. Although we did not quantify light interception by dominant species, this is consistent with the observed differences in SLA, toughness, δ^13^C, and thickness among treatments (Figure 2). Higher SLA, more negative δ^13^C values, and lower leaf thickness and toughness, as observed in mixtures, are indeed typical response patterns to more shaded conditions (Pérez‐Harguindeguy et al., 2013). Furthermore, differences in leaf traits between oak monocultures and oak‐pine mixtures were clearly shown on the first PCA axes (Figure 3) while leaf traits in oak‐birch mixtures were more similar to leaf traits in monocultures. Given the canopy shape and foliar content of birch and pine trees, one can hypothesize that even though the two species have similar mean heights, solar radiation is more intercepted by pine trees (which are more opaque). Light could thus be one factor controlling leaf traits of oaks growing below the canopy of taller neighbors, but it was probably not the only one. Furthermore, our results do not support the hypothesis that variability in leaf traits among plots with different specific compositions was driven by differential response to water stress (Walter et al., 2011; Forey et al., 2016) as irrigation had no effect on leaf traits, neither in univariate nor in multivariate analyses. It must be acknowledged that we applied irrigation only few weeks before traits were measured, and it cannot be excluded that stronger effects will emerge in the future. Here, we can only speculate on the mechanisms responsible for such differences among mixtures, but it is likely that litter composition or understory vegetation differentially also affected oak traits in monocultures and oak‐birch or oak‐pine mixtures (Nickmans, Verheyen, Guiz, Jonard, & Ponette, 2015).

#### 4.1.2 Leaf traits predicted only a limited fraction of insect herbivory

As oak neighbor identity affected variability in oak leaf traits, we expected that particular traits might be the functional link between oak neighbor identity and herbivory. This would have been in accordance with recent work showing trait‐mediated effects of heterospecific plant neighbors on insect herbivores and herbivory (Halpern et al., 2014; Ohgushi & Hambäck, 2015; Kos et al., 2015; Kostenko et al. 2017). Our results weakly support this prediction. Indeed, only leaf C:N ratio had an effect on herbivory (Figure 5), yet this trait did not vary with oak neighbor identity (Figure 2). Individually, none of the other traits measured in early season had a significant effect on herbivory. However, traits may have had a combined effect on herbivory as suggested by the multivariate analysis. This effect was, however, complex and explained only 8% of total variability in herbivory.

Although our results partially support the idea that tree species composition had indirect trait‐mediated effects on overall insect herbivory, they should be considered with caution. Previous studies reported mixed evidence for such a relationship between plant diversity, leaf traits, and herbivory (Moreira et al., 2014; Abdala‐Roberts, 2016; Kostenko et al. 2017). Here, because we measured total chewing damage, we cannot dismiss the fact that the overall pattern obscures species‐specific variability in herbivore response to leaf traits (Heiermann & Schütz, 2008; Plath et al., 2011), which would deserve more investigation.

### 4.2 Predation barely changed with oak neighbor identity

The enemies’ hypothesis posits that more species‐rich forest plots should shelter a greater diversity or abundance of predators than monocultures, enhancing potential biological control of herbivores. In particular, mixing conifer and deciduous tree species at both forest stand and landscape scales is expected to increase insectivorous predator abundance (Charbonnier et al., 2016; Oxbrough, García‐Tejero, Spence, & O'Halloran, 2016), with cascading effects on pest control (Felton et al., 2016; Giffard, Barbaro, Jactel, & Corcket, 2013). Although the enemies’ hypothesis has received some empirical support in grasslands and agro‐ecosystems (Andow, 1991; Langellotto & Denno, 2004; Letourneau et al., 2011; Scherber et al., 2010), its validity for forest is still debated (Letourneau, Jedlicka, Bothwell, & Moreno, 2009; Muiruri et al., 2016; Riihimäki et al., 2004; Schuldt et al., 2011). In addition, it must be noticed that the use of dummy caterpillars underestimates the importance of top‐down processes as it does not capture the effect of other natural enemies such as spiders or parasitoids which are known to, respectively, respond to structural and chemical complexity of their habitat (Kostenko et al., 2015; Langellotto & Denno, 2004).

### 4.3 Irrigation had no direct effect on herbivory but indirectly changed predation

*Although predawn leaf water potentials (*ψ_*w*_ *) and carbon isotope values (*δ^*13*^ *C) confirmed that irrigation alleviated water stress, neither leaf traits nor insect herbivory was influenced by irrigation*. However, water stress varied among oaks with different neighbors as indicated by the more negative ψ_*w*_ and less negative δ^13^C in oak‐pines mixtures, both indicating stronger water stress. This could be due to pines being much taller than oaks and exploiting a greater amount of water. Such an effect of interspecific competition for water may have been less pronounced with birches that lose leaves during summer, while pines kept their needles.

The lack of effect of irrigation on leaf traits and herbivory was unexpected, as the influence of drought on leaf functional traits has been known for decades (Chaves, Maroco, & Pereira, 2003). However, ψ_*w*_ in the rainfall only plots did not reach very negative values during the 2015 growing season. They remained above −0.5 MPa and the difference in ψ_*w*_ suggests that during the 2015 growing season, unirrigated trees were subject to low water stress. Yet, the response of herbivores may vary nonlinearly with stress intensity (Gutbrodt, Dorn, & Mody, 2011; Jactel et al., 2012). Therefore, we cannot exclude that the lack of effect of irrigation on leaf traits and herbivory was due to low drought intensity.

Moreover, irrigation started only a few weeks before trait measurements. This period was enough to influence soil water availability in the rainfall only versus irrigation plots (as seen in ψ_*w*_ values, Fig. S1), but it may not have been long enough, and the soil water deficit may not have been severe enough, to significantly influence leaf morphological and physiological properties. This later assumption is also supported by the fact that many temperate *Quercus* species have preformed growth of shoots in buds (Fontaine et al., 2000): The organogenesis period for the first growth unit ranges approximately from August to October of the previous year (Fontaine et al., 2000), whereas the elongation periods range from March to September of the current year. The influence of changing environmental conditions in spring on *Quercus* leaf traits may have thus been lower than for species in which leaf organogenesis is directly occurring in spring. This explanation is also consistent with the fact that changes in herbivory‐related leaf traits involving cell division and differentiation (such as those we measured: SLA, LDMC, toughness) take longer than changes in leaf chemistry involving the production of secondary metabolites (Herms & Mattson, 1992; Moreira et al., 2014) and may contribute to explaining why we did not detect any significant effect of irrigation on leaf structural traits.

Predation was the only response variable that was influenced by drought, with greater predation in irrigated plots, especially by rodents and arthropods. This may result from understorey vegetation being denser and more stratified in irrigated plots (*personal observations*), potentially resulting in larger niche opportunities, higher prey availability, or lower top‐down predation risk by apex predators (Figure 4a). Similar effects of structural complexity have been observed by Poch and Simonetti (2013) who detected higher predation rates in forest plantations with a more developed understorey. Further investigation will be needed to tease apart the potential direct effects of irrigation on herbivory and indirect effects mediated by predation.

## 5 Conclusion

We did not find strong support to some of the most commonly proposed hypotheses to explain herbivory patterns, notably the apparency hypothesis. We found no clear support for the enemies’ hypothesis. We found only partial support for the emerging idea that associational effects could be mediated by change in leaf traits relevant to insect herbivory. Although we could only speculate on the mechanisms linking tree species composition, variability in leaf traits, and herbivory, our results suggest that indirect trait‐mediated effects (including secondary metabolites) will be a key aspect to consider in the future studies addressing associational effects of plant diversity on higher trophic levels.

## Conflict of interest

None declared.
