# Supplementary material for: Bottom‐up and top‐down effects of tree species diversity on leaf insect herbivory
Source: Ecol Evol. 2017 Apr 9;7(10):3520–31. doi: 10.1002/ece3.2950 (PMC5433970; doi:10.1002/ece3.2950)
Supplement: Supplementary file 1 [file ECE3-7-3520-s001.docx]

**Bottom-up and top-down effects of tree species diversity on leaf insect herbivory**

**– Electronic supplementary material –**

**Authors:** Bastien CASTAGNEYROL^1^, Damien BONAL^2^, Maxime DAMIEN^3^, Hervé JACTEL^1^, Céline MEREDIEU^1^, Evalyne W. MUIRURI^4,5^, Luc BARBARO^1,6^

**Affiliations:** ^1^ BIOGECO, INRA, Univ. Bordeaux, 33610 Cestas, France

^2^ INRA, UMR EEF Université de Lorraine / INRA, 54280 Champenoux, France

^3^ Univ. Rennes1, UMR CNRS 6553, ECOBIO, F-35042 Rennes, France

^4^ School of Biological Sciences, Royal Holloway University of London, Egham, Surrey TW20 0EX, UK

^5^Department of Biosciences, Durham University, Stockton Road, Durham, DH1 3LE

^6^Dynafor, INPT, EI Purpan, INRA, Univ. Toulouse F-31320, Auzeville, France

**Correspondence** Bastien CASTAGNEYROL, INRA – UMR 1202 BIOGECO, 69 route d'Arcachon, 33612 Cestas (France)

☎ +33(0)5 57 12 27 30 ✉ [bastien.castagneyrol@HYPERLINK "mailto:bastien.castagneyrol@pierroton.inra.fr"inra.fr](mailto:bastien.castagneyrol@pierroton.inra.fr)

**Figure S1:** The Orphee experiment.

**Figure S2:** Effects of irrigation and tree species composition on predawn water potential in oaks.

**Table S1:** Summary of LMMs testing the effects of leaf traits, insectivory, plot irrigation and oak neighbour identity on early season insect herbivory.

**Figure S1**: The ORPHEE experiment (see also [www.facebook.com/orpheeexperiment](http://www.facebook.com/orpheeexperiment))


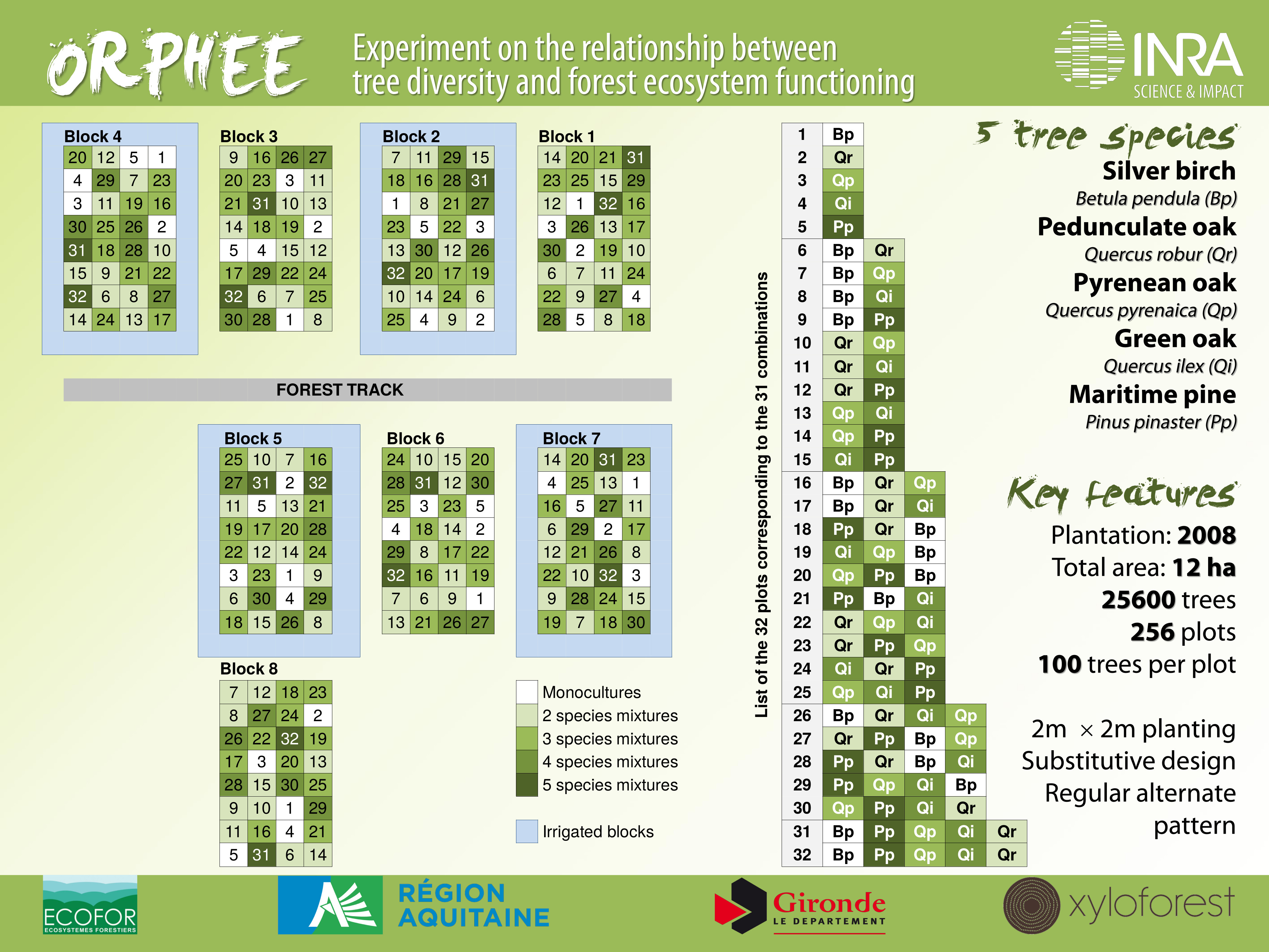


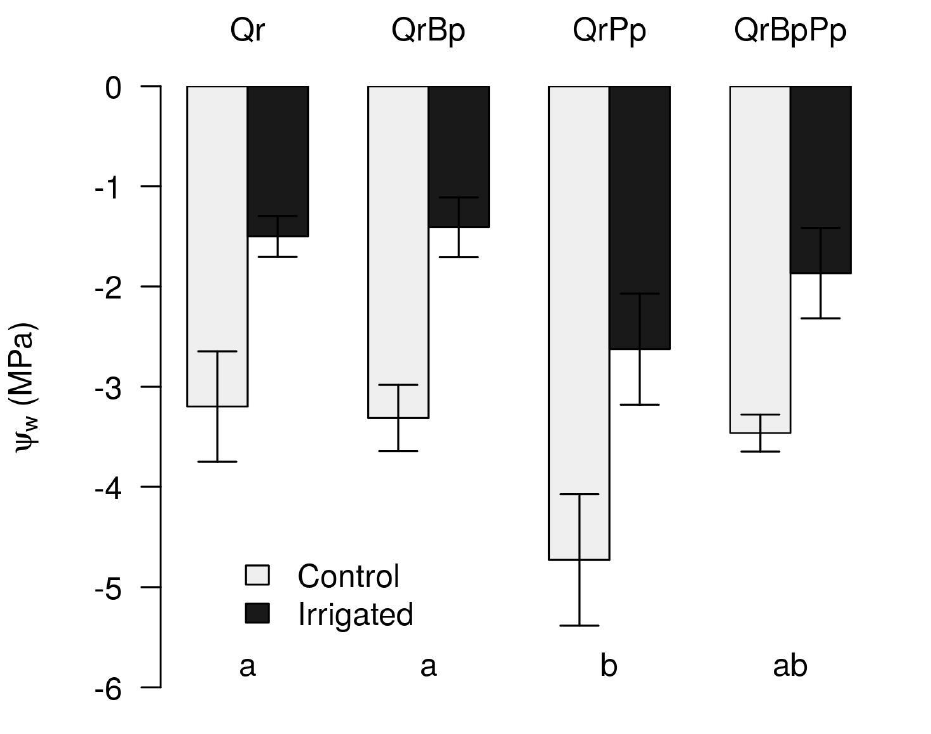


**Figure S2: Effects of irrigation and tree species composition on predawn water potential in oaks.** Same letters below bars indicate non significant differences. Upper case and lower case letters are for irrigation (I) and composition (C) effects, respectively. Qr: *Quercus robur*, QrBp: *Q. robur*+ *Betula pendula*, QrPp: *Q. robur* + *Pinus pinaster*, QrBpPp: *Q. robur* + *B. pendula* + *P. pinatser*.

**Table S1:** Summary of LMMs testing the effects of leaf traits, insectivory, plot irrigation and oak neighbour identity on early season insect herbivory.

| **Predictor** | ***F*-value (df)** | ***P*-value** |
| --- | --- | --- |
| LDMC | 0.26 (1, 61.5) | 0.611 |
| Thickness | < 0.01 (1, 55) | 0.984 |
| Toughness | 0.32 (1, 23.8) | 0.576 |
| SLA | 1.49 (1, 65.6) | 0.227 |
| Insectivory | 0.32 (1, 44.0) | 0.578 |
| Irrigation | 0.02 (1, 5.60) | 0.897 |
| Neighbour | 0.69 (3, 55.3) | 0.506 |
| Irrigation × Neighbour | 0.68 (3, 53.6) | 0.567 |
